# Apexification with a New Intra-Canal Medicament: A Multidisciplinary Case Report

**Published:** 2012-08-01

**Authors:** Adriana de Jesus Soares, Juliana Yuri Nagata, Renato Corrêa Viana Casarin, José Flávio Affonso de Almeida, Brenda Paula Figueiredo de Almeida Gomes, Alexandre Augusto Zaia, Caio Cezar Randi Ferraz, Francisco José de Souza-Filho

**Affiliations:** 1. Department of Restorative Dentistry, Endodontics Area, State University of Campinas-UNICAMP, Piracicaba, SP, Brazil; 2. Periodontics Division, Paulista University, São Paulo, Brazil

**Keywords:** Apexification, Endodontics, Calcium Hydroxide, Periodontics, Tooth Injuries

## Abstract

Dental trauma generally requires multidisciplinary planning and treatment for good prognosis. When immature teeth are traumatized to a degree where pulp necrosis ensues, the objective of root canal treatment should be apexogenesis and root maturation. Apexification of the root is the conventional choice, which involves cleaning the canal and filling it with a temporary medication that stimulates the formation of a calcific apical barrier. Dental Trauma Service of Piracicaba Dental School, State University of Campinas (UNICAMP), Brazil employs a dressing for apexification treatments with calcium hydroxide, chlorhexidine gel 2% and zinc oxide. This paper reports the case of a dental trauma of the maxillary central incisors and subluxation on teeth 11, 12 and 21 that were treated with multidisciplinary collaboration (Endodontics, Periodontology and Operative Dentistry) to improve prognosis. After five-years there were no pathological conditions and the teeth showed every evidences of success.

## Introduction

Dental trauma may be considered a multifactorial health problem worldwide [1][2] that frequently requires multidisciplinary treatment planning, mainly with the participation of Endodontic, Operative Dentistry and Periodontology. Literature has showed the importance of integrated planning to improve quality of life of the patient [3].

Dental trauma happens most frequently in young patients, who generally present immature teeth (with open apex) [4]. In this case, if the pulp is necrotic, treatment involving root maturation must be considered. It can be achieved by preventing the contamination of the root canal and with the dressing of the canal with a material capable of stimulating the mineralized barrier.

Apexification is the conventional treatment involving cleaning the canal and filling it with a temporary medication that stimulates the formation of a calcified tissue in the apex. With the observation of clinic and radiographic evidence of apex closure, the intracanal medication may be removed and a permanent obturation with gutta-percha can be placed. Several studies have followed this protocol, with materials such as calcium hydroxide [5][6][7][8][9][10]. The time required for apical barrier formation may be as long as 20 months using Ca(OH)_2_. Age and presence of symptoms or perirradicular radiolucencies may affect the time needed to form an apical barrier [11].

Refreshing the Ca(OH)_2_ paste usually takes place every three months, which requires multiple visits with heavy demands on patients and operators, inevitable clinical costs, and the increased risk of tooth fracture using Ca(OH)_2_ since many dressing changes are necessary till the formation of a calcified barrier [12][13]. These drawbacks encourage a search for alternatives for the use of calcium hydroxide. A promising alternative to achieve the goal of apexification is the mixture of this well-known medication (calcium hydroxide) with chlorhexidine gel 2% and zinc oxide that may be a low-cost, easy-to-use, high radiopacity, no-need-for-periodic-exchanges, and mineralizing material [14]. Dental Trauma Service of Piracicaba Dental School, State University of Campinas (UNICAMP) has been using this mixture dressing in dental trauma cases such as avulsion and immature teeth. These cases are clinically and radiographically followed-up every three months, without the requiring medicament changes. This paper reports a case of apexification by using a multidisciplinary approach and a mixture of calcium hydroxide with chlorhexidine gel 2% and zinc oxide powder as root canal medicament.

## Case Report

A 9-year-old female patient came to the Dental Trauma Service of the Piracicaba Dental School, State University of Campinas, Piracicaba, SP, Brazil, with multiple traumatized permanent maxillary incisors. The patient and her sponsor reported that she had a fall fifteen days before and had injured her teeth. She was referred by her general dental practitioner to the Dental Trauma Service of the Piracicaba Dental School.

**Figure 1:**
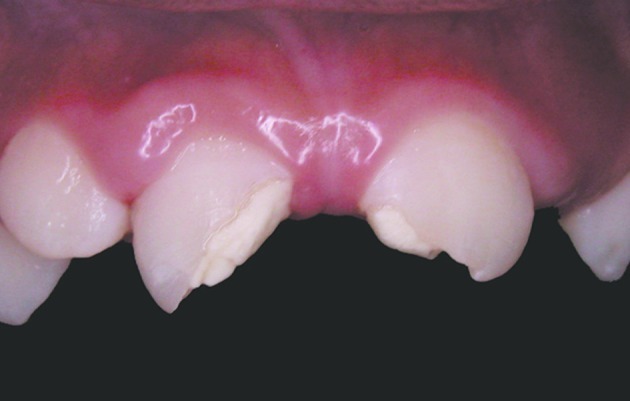
Initial clinical photographic view

**Figure 2:**
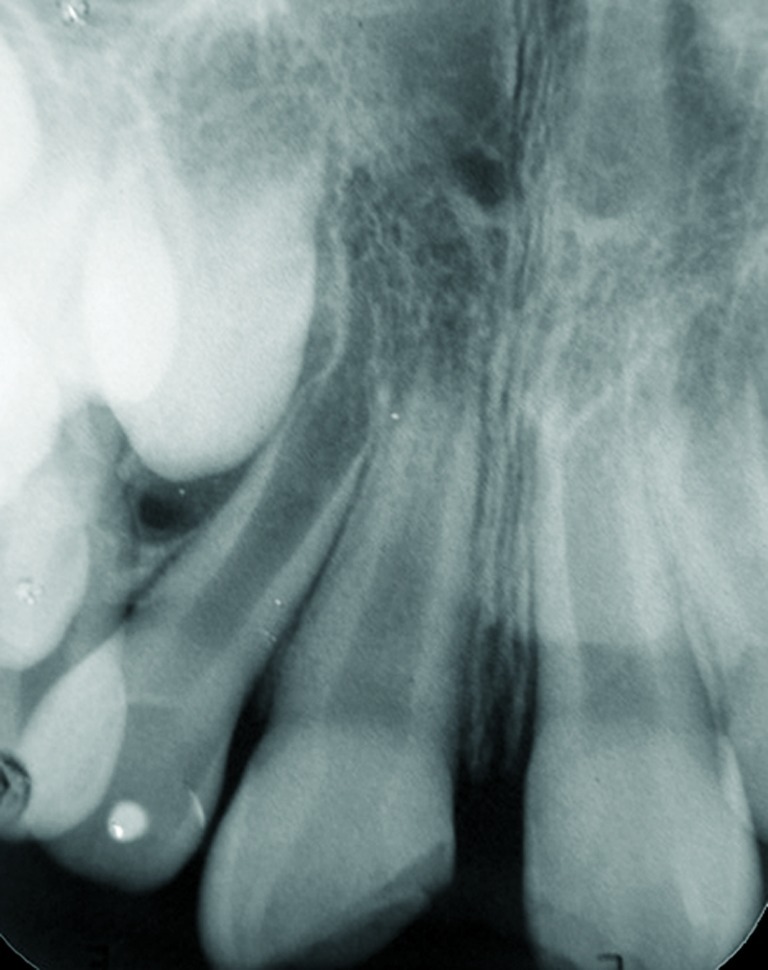
Initial periapical radiograph revealing incisors’ incomplete root formation

At the first visit, clinical examination (including extra-oral examinations, pulp tests, palpation and percussion) of all the affected incisors showed questionable vital diagnoses by cold-thermal pulp test (Endo Frost, Roeko, Langenau, Germany), and sensitivity to the percussion and palpation periapical tests (Figure 1). Radiographic examination detected no alveolar root and bone fractures, and enlargement of periodontal ligaments of the right central and lateral incisors. Moreover, the root formations of the affected teeth were incomplete (Figure 2). These observations (clinical and radiographic data) led to the diagnosis that the right central incisor suffered extrusive luxation and a subgingival enamel and dentinal crown fracture. The left central incisor had enamel and dentinal fracture, the lateral right incisor was damaged by a subluxation. As the vitality tests were questionable, no endodontic procedure was carried out in this visit. After fifteen days, diagnostic tests were performed again; these showed that the right central incisor was non-vital and the right lateral incisor and left central incisor were vital. Endodontic treatment with apexification for the right central incisor was planned.

**Figure 3:**
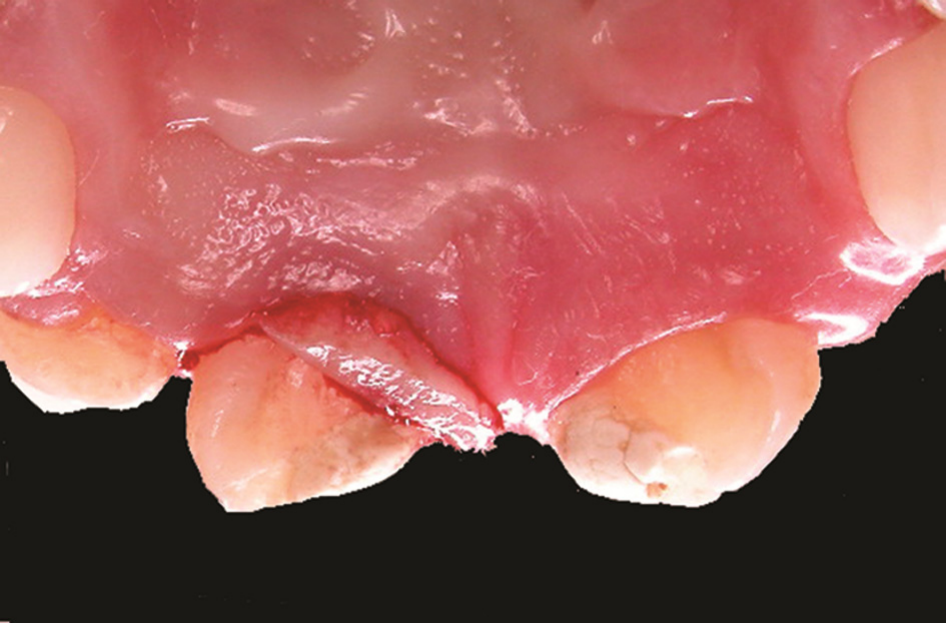
Palatinal view of the gingivectomy

**Figure 4:**
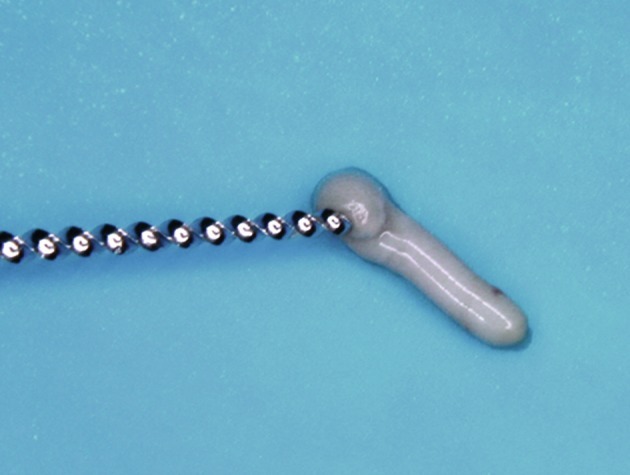
Extirpated pulp tissue revealing its necrotic aspect

**Figure 5:**
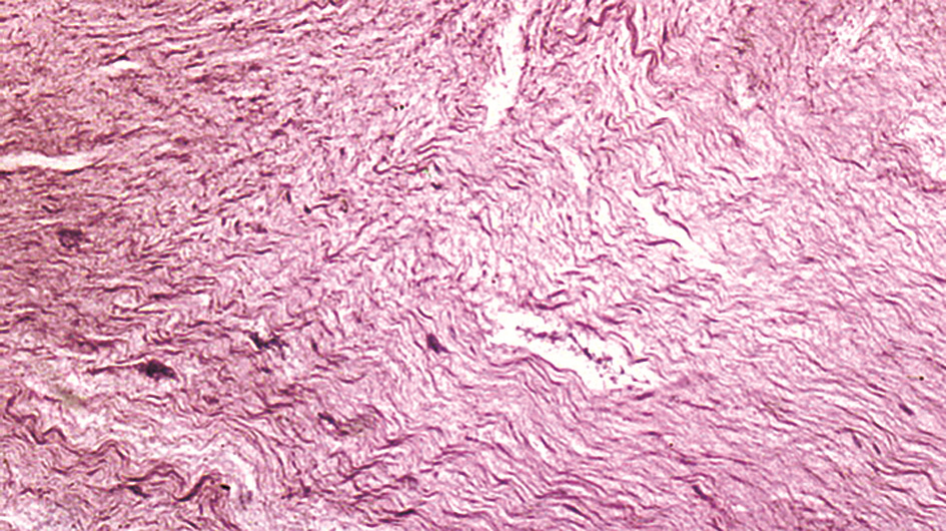
Histological analysis of pulp tissue showing necrotic tissue, characterized by loose conjunctive tissue in degeneration stage, absence of cellular nucleus and disorganization of collagen fibers (Hematoxylin/Eosin, 200×)

**Figure 6:**
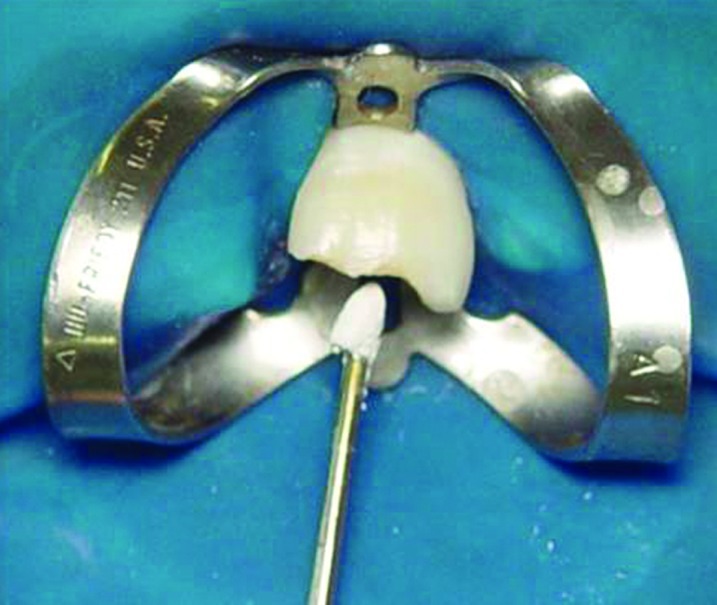
Intracanal medication inserted with condenser

Clinically, we observed that absolute isolation would be difficult and that we could not visualize the end of the coronal fracture because of the incomplete tooth eruption. In order to achieve an adequate environment to endodontic and restorative procedures, a gingivectomy was performed with removal of 2-3 mm of keratinized palatal mucosa around the maxillary central incisors (Figure 3). Afterward the tooth was treated by first creating a coronal access, extirpating the necrotic pulp (Figure 4 and Figure 5), and then performing manual (Dentsply Maillefer, Balaigues, Switzerland) crown-drown instrumentation, cleaning and shaping of the canal with constant irrigation with 2% chlorhexidine gel (Endogel, Itapetininga, SP, Brazil) and physiological solution. Afterwards, the root canal was dried and a solution of 17% EDTA (Odhacam/Dentsply, Rio de Janeiro, RJ, Brazil) was applied into the root canal for three minutes. The obturation paste was prepared from the mixture of calcium hydroxide (Biodinâmica, Ibiporã, PR, Brazil), 2% chlorexidine gel and zinc oxide (Biodinâmica, Ibiporã, Paraná, Brazil) in a 2:1:2 proportion. It was inserted into the root canal to stimulate the apical closure (Figure 6) and the tooth was coronally sealed with coltisol and composite resin.

**Figure 7:**
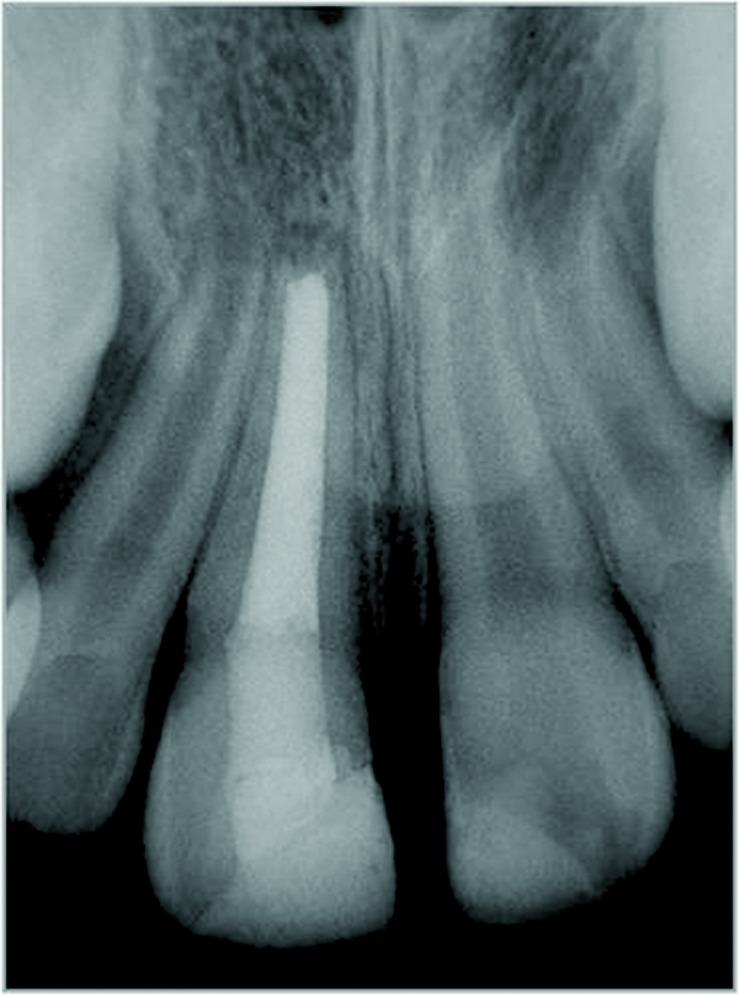
Six-month follow-up radiograph reveals a mineralized bridge in the apical region

**Figure 8:**
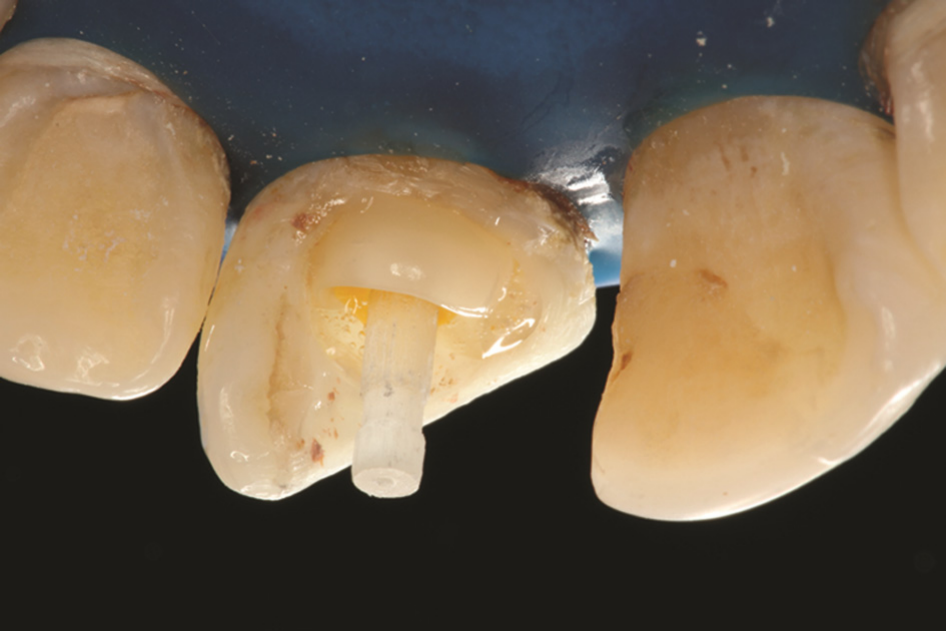
Palatinal view of modeled fiber glass post

**Figure 9:**
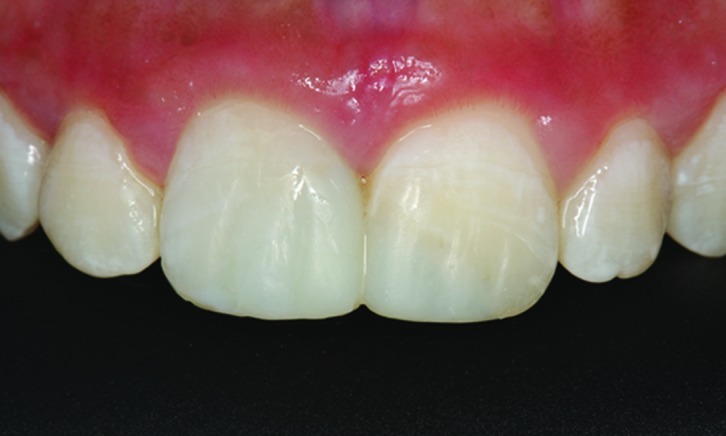
Clinical aspect of the restored teeth afterward

**Figure 10:**
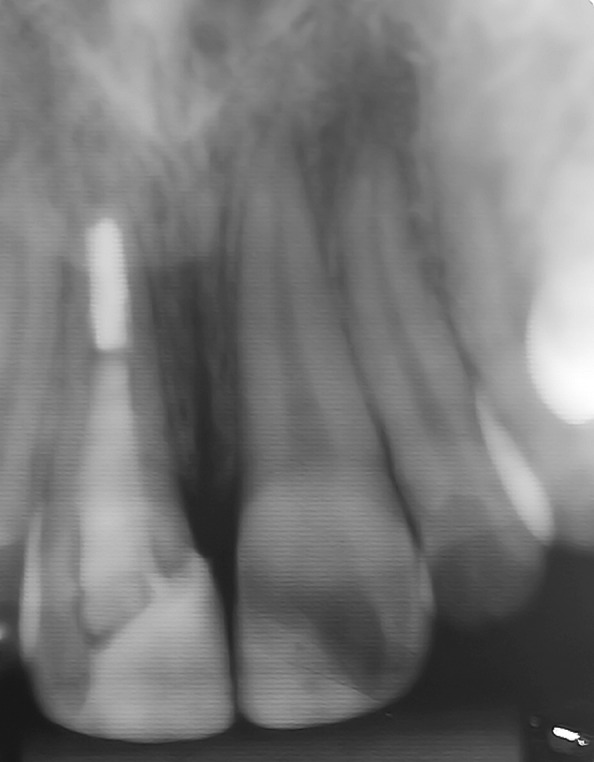
Final radiograph after aesthetic restoration

Patient was recalled for periodic visits (every three months) for clinical and radiographic evaluation. The paste did not need to be exchanged for nine months. After nine months, apical closure (Figure 7) was achieved without periapical symptoms and mobility, allowing root canal obturation with gutta-percha. After this, follow up visits were performed every year in 2007, 2008, 2009 and 2010. At the 2011 follow-up appointment, clinical examination observed an unsatisfactory restoration on maxillary central incisors. Maxillary right central incisor was re-restored with fiberglass post (Exacto translúcido, Angelus™, Londrina, Brazil) which was modeled with composite resin and conditioned with silane, acid, primer, catalyst and activator (Angelus, Londrina, Paraná, Brazil) (Figure 8). After cementation, the crown was restored with composite resin (4 Seasons Ivoclar Vivadent, New York, USA). The final aesthetic appearance of the patient may be seen in Figure 9 and Figure 10.

## Discussion

The majority of dental trauma patients require multidisciplinary cooperation; this includes young patients who have not finished tooth development. Literature has reported multidisciplinary planning [3][15][16], showing how adequate integrated treatment planning, coordination, and execution are necessary for the proper management of complex cases. In the presented case, the patient regained her esthetic and function due to cooperation of Endodontics, Operative Dentistry, and Periodontology departments.

Unlike the apexification related in this article, recently, revascularization has been studied as an alternative treatment in some cases of incomplete root formation, because it stimulates the thickness and apical closure of immature teeth [17][18]. However, revascularization may have potential for clinical and biological complications. Amongst them, crown discoloration [19], development of resistant bacterial strains and allergic reaction to the intracanal medication [20]. Moreover, the mechanism of pulp revascularization, the type of tissue that has been developed on root canal walls and the clinical consequences of long period follow-up are still unclear. Considering these aspects, in the present case a more predictable treatment (apexification) was the treatment of choice.

The protocol of the Dental Trauma Service of Piracicaba Dental School (obturation paste without exchanges and coronal sealing) has shown clinical and radiographic success in traumatized immature teeth with apical barrier formation [21]. A clinical study with traumatized immature teeth showed apical closure after nine months and reduction of all the symptoms and signs after carrying out treatment according to this protocol [21]. It has been suggested that the hydroxyl ions of this obturation paste have a good diffusion on dentinal tubules [22], and in thirty days, no pH alterations have been observed with this medication [23]. Another advantage is that it does not need to be changed during the period that apexification is occuring as no dissolution is observed radiographically during the control visits. The fact that medicaments do not have to be removed is advantageous; for example the prevention of infection during replacement of the medication and the reduced time necessary for the apical barrier formation. Moreover, radiographs showed that the material did not undergo dissolution. It is likely that after nine months, only zinc oxide was present, since Ca(OH)_2_ should have undergone complete dissolution. The presence of zinc oxide should have worked as an inert sealing material, preventing contamination and allowing apical repair and barrier formation. The absence of a good sealing and the presence of radiographic and clinical symptoms may indicate the replacement of the medication. The composition, mechanism of action and long term follow-up of cases treated with this intracanal dressing should be more studied.

## Conclusion

The apexification of necrotic traumatized teeth is still the treatment of choice in cases with incomplete root formation. The protocol used also allowed periapical repair. In addition, multidisciplinary action greatly contributed to the successful outcome of this case.
